# Functional testing is a complementary tool for the diagnosis of vaginitis

**DOI:** 10.1186/s12905-024-03035-w

**Published:** 2024-04-06

**Authors:** Danqin Feng, Fuhui Zhang, Jianguo Cai, Yansheng Zhang, Honghong Yan, Yichi Yang, Hongxiu Zhong, Huiming Ye

**Affiliations:** 1 Department of Laboratory Medicine, Fujian Key Clinical Specialty of Laboratory Medicine, Women and Children’s Hospital, School of Medicine, Xiamen University, Xiamen, China; 2 Department of Social Medicine, Graduate School of Medicine, Hirosaki University, Hirosaki, Japan; 3 Department of Obstetrics and Gynecology, Women and Children’s Hospital, School of Medicine, Xiamen University, Xiamen, China

**Keywords:** Vaginitis, Functional tests, Morphological tests, Vaginal microbiota evaluation

## Abstract

**Objective:**

Vaginal microbiota evaluation is a methodology widely used in China to diagnose various vaginal inflammatory diseases. Although vaginal microbiota evaluation has many advantages, it is time-consuming and requires highly skilled and experienced operators. Here, we investigated a six-index functional test that analyzed pH, hydrogen peroxide (H_2_O_2_), leukocyte esterase (LEU), sialidase (SNA), β-glucuronidase (GUS), and acetylglucossidase (NAG), and determined its diagnostic value by comparing it with morphological tests of vaginal microbiota.

**Materials and methods:**

The research was conducted using data extracted from the Laboratory Information System of Women and Children’s Hospital. A total of 4902 subjects, ranging in age from 35.4 ± 9.7 years, were analyzed. During the consultation, a minimum of two vaginal swab specimens per patient were collected for both functional and morphological testing. Fisher’s exact was used to analyze data using SPSS.

**Results:**

Of the 4,902 patients, 2,454 were considered to have normal *Lactobacillus* morphotypes and 3,334 were considered to have normal dominant microbiota. The sensitivity and specificity of H_2_O_2_-indicating *Lactobacillus* morphotypes were 91.3% and 25.28%, respectively, while those of pH-indicating *Lactobacillus* morphotypes were 88.09% and 59.52%, respectively. The sensitivity and specificity of H_2_O_2_-indicating dominant microbiota were 91.3% and 25.3%, respectively, while those of pH-indicating dominant microbiota were 86.27% and 64.45%, respectively. The sensitivity and specificity of NAG for vulvovaginal candidiasis were 40.64% and 84.8%, respectively. For aerobic vaginitis, GUS sensitivity was low at 0.52%, while its specificity was high at 99.93%; the LEU sensitivity and specificity values were 94.73% and 27.49%, respectively. Finally, SNA sensitivity and specificity for bacterial vaginosis were 80.72% and 96.78%, respectively.

**Conclusion:**

Functional tests (pH, SNA, H_2_O_2_, LEU) showed satisfactory sensitivity for the detection of vaginal inflammatory diseases. However, these tests lacked specificity, making it difficult to accurately identify specific pathologies. By contrast, NAG and GUS showed excellent specificity in identifying vaginal inflammatory diseases, but their sensitivity was limited. Therefore, functional tests alone are not sufficient to diagnose various vaginal inflammatory diseases. When functional and morphological tests are inconsistent, morphological tests are currently considered the preferred reference method.

Reproductive tract infections are remarkably common. In China, approximately 40% of women who consult a physician suffer from a reproductive tract infection. Such infections have become a major social and public health problem throughout the world [1, 2], and the diagnosis of this type of infection is therefore vital. Bacterial vaginosis (BV), *Trichomonas vaginitis* (TV), vulvovaginal candidiasis (VVC), and aerobic vaginitis (AV) are the four most common etiologies of vaginitis. To combat these diseases, the Committee on Infectious Diseases, Division of Obstetrics and Gynecology, Chinese Medical Association, has developed a novel vaginal microecological detection instrument - vaginal microbiota evaluation. Vaginal microbiota evaluation helps to enhance the accuracy of diagnosis, particularly for the diagnosis of mixed reproductive tract infections, and to optimize the selection of clinical therapies, thus promoting the restoration of vaginal microecological balance [1, 3, 4]. Vaginal microbiota evaluation involves the implementation of the Nugent score and Donders’ score. The Nugent score for the diagnosis of BV was observed to be reproducible across different centers and microbiologists, and included a permanent record of the patient specimen used for diagnosis [5]. Because many infectious agents unrelated to BV, such as aerobic vaginal pathogens, are known to be associated with perturbation of the lactobacillary flora [6], the Donders’ score is also needed for vaginal microbiota evaluation. In resource-limited primary healthcare facilities, the benefits of vaginal microecological evaluation are often mitigated by lengthy Gram staining microscopic procedures, high staff workload, and extended diagnostic and treatment time frames. Therefore, there is a need to explore alternative methods for rapid and comprehensive evaluation of vaginal microecology in hospitals [7].

In China, a significant number of manufacturers have transitioned to functional test production. According to the manufacturer’s instructions (GDFDA 20,162,400,158), a higher concentration of hydrogen peroxide (H_2_O_2_) is typically associated with a higher number of *Lactobacillus* spp. Sialidase (SNA) can be positively identified in anaerobic bacteria, while some aerobic bacteria show β-glucuronidase (GUS) activity, and some *Candida albicans* also show N-acetylglucosaminidase (NAG) positivity. Notably, the vaginal mucosa is particularly susceptible to damage and inflammation during the proliferation of pathogenic microorganisms, leading to the release of leukocyte esterase (LEU). Functional tests for vaginitis, such as sialidase activity, are common methods used to detect vaginitis. In this study, we aimed to validate the usefulness of commercially available functional tests for vaginitis in comparison with morphological tests.

## Methods

### Study design and setting

**Fig. 1:**
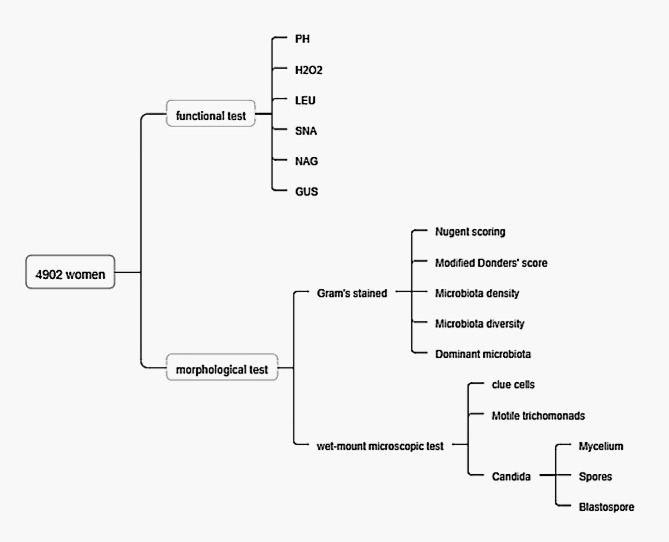
Flow chart of the research process. One swab was used for functional tests and the other for morphological tests. The functional tests measured the concentrations of GUS (β-glucuronidase), NAG (acetylglucosidase), SNA (sialidase), LEU (leukocyte esterase), and H_2_O_2_ (hydrogen peroxide), as well as the pH. The morphological test comprised a Gram stain and a wet-mount microscopic test

Data were collected from the “Laboratory Information System” of Women and Children’s Hospital. From August 2021 to October 2022, a total of 4902 patients were presented to the Department of Gynecology and Obstetrics, Medical Women and Children’s Hospital, School of Medicine, Xiamen University, China, for vaginal microbiota evaluation. This study was authorized to receive a waiver for informed consent in the “ethical approval and consent to participate” phase of the research process by the Ethical Council of Human Research at Xiamen Maternal and Child Health Care Hospital (KY-2023-087-K01). Women who had not engaged in sexual intercourse, tub bath, vaginal lavage, or used topical medication within the previous 24 h, and were 35 ± 9.7 years of age were eligible to complete the vaginal microbiota evaluation during their consultation. Typically, the laboratory collected two vaginal swab specimens from each patient. One swab was used for functional tests and the other was used for morphological tests (Fig. 1).

### Functional tests

A vaginal swab specimen was placed in a tube along with 400 mL of diluent. After the vaginal swab was eluted with the appropriate eluent, samples were analyzed using the LTS-V800 vaginal secretions analyzer (Zhuhai Lituo Biotechnology Co., Ltd).The six-index combined detection method (Zhuhai Lituo Biotechnology Co., Ltd) was used according to the manufacturer’s instructions. The six indicators were the pH value, and the β-glucuronidase (GUS), acetylglucossidase (NAG), sialidase (SNA), leukocyte esterase (LEU), and hydrogen peroxide (H_2_O_2_) concentrations.

### Morphological tests

Ten drops of saline were applied to the swab with vaginal secretions, and after the secretion had been eluted, the eluant was dripped onto two glass sides. One was heat fixed and Gram stained to assess the Nugent score [5](Table 1), modified Donders’ score [8, 9](Table 2), microbiota density, microbiota diversity, dominant bacteria, and pathogenic microorganisms. The other was a wet-mount microscopic test for clue cells and motile trichomonads, with potassium hydroxide for the detection of candida (mycelia, spores, and blastospores). An alteration in microbiota density would suggest changes in the total biomass of bacteria in the microbial ecosystem (Table 3). Microbiota diversity was calculated to show the range of bacterial species in the smear (Table 3). Dominant bacteria were those with the largest biomass density among the microbiota, and such organisms play an essential role in the host’s physiology and pathology [9]. Both wet-mount and Gram-stained smears were examined by trained professional technicians.

**Table 1:** Nugent scoring for BV^a^ [5].

Score^b^	Lactobacillus	Gardnerella/Bacteroides spp.	Curved Gram-Variable Rods
0	4+	0	0
1	3+	1+	1 + or 2+
2	2+	2+	3 + or 4+
3	1+	3+	-
4	0	4+	-

^a^ Morphotype score was the average number of bacterial cells per oil immersion field. Total score = *Lactobacillus* + *Gardnerella* /*Bacteroides* spp. + curved gram-variable rods

^b^ Quantification of each individual score: 0, no morphotypes present; 1+, < 1 morphotype present; 2+, 1 to 4 morphotypes present; 3+, 5 to 30 morphotypes present; 4+, 30 or more morphotypes present

^c^ A score of 0–3 was considered “normal”, 4–6 as considered an “intermediate” vaginal microbiota, and 7–10 was considered BV.

**Table 2:** Aerobic vaginitis modified Donders’ score [8, 9]

Score	LBG (1000×)	Number of Leukocytes (400×)	Background Flora (1000×)	Proportion of PBC (400×)
0	I, IIa	*≤* 10/HPF	Unremarkable or cytolysis	< 1%
1	IIb	> 10/HPF and *≤* 10/epithelial cells	Small coliform bacilli	*≥* 1% and *≤* 10%
2	III	> 10/epithelial cells	Cocci or chains	> 10%

^a^ The number of leukocytes and the proportion of parabasal epitheliocytes (PBC) were evaluated by light microscopy (400*×* magnification). Lactobacillary grades (LBG) and background flora were evaluated by oil immersion (1000*×* magnification).

^b^ Aerobic vaginitis diagnosis was mainly based on clinical characteristics and identified by a Donders’ score of ≥ 3 points

**Table 3:** Grading standards of microbiota density and microbiota diversity [9]

Grading standards	Microbiota density (the average number of bacteria)^a^	Microbiota diversity (bacterial species)^a^
Grade I(+)	1 ∼ 9	1 ∼ 3
Grade II(++)	10 ∼ 99	4 ∼ 6
Grade III(+++)	100 ∼ 999	7 ∼ 9
Grade IV(++++)	≥ 1000	≥ 10

^a^ Oil microscope, magnification 10 × 100.

### Statistical analysis

Quantitative data were analyzed using SPSS version 24.0 and interpreted according to the interquartile range. Fisher’s exact test was used to identify significant associations, with p-values less than 0.05 considered significant. The area under the receiver operating characteristic (ROC) curve measured the pH performance using appropriate software.

## Results

In this study, of the 4,902 patients analyzed, 2,454 were found to have normal *Lactobacillus* morphotypes(30 or more morphotypes present) and 3,314 patients had normal dominant microbiota. Furthermore, 534 suffered from VVC; 1,916 patients were evaluated as having an AV score of ≥ 3 points, and 332 patients were diagnosed as having BV (Nugent score ≥ 7).

**Fig. 2:**
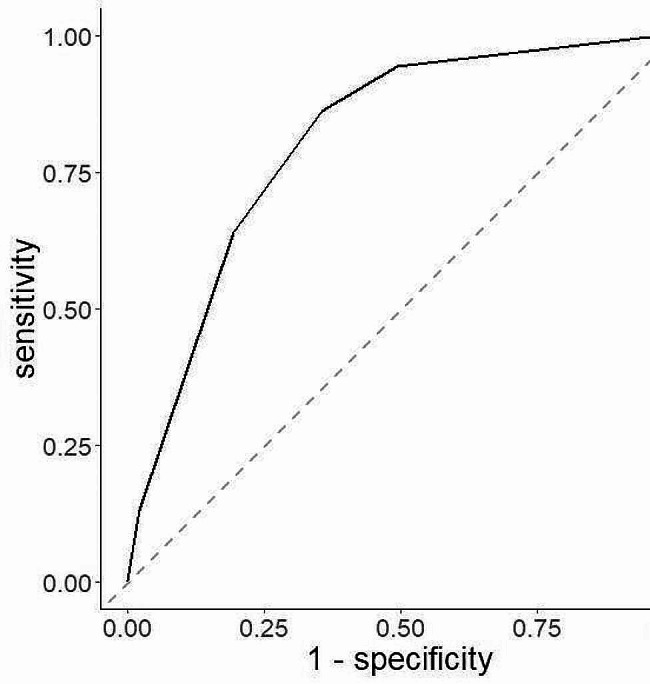
ROC plot with pH as an independent variable and lactobacillus morphotypes as a dependent variable. The horizontal axis represents 1 – specificity, while the vertical axis represents sensitivity. The AUC for lactobacillus morphotypes was 0.83

**Fig. 3:**
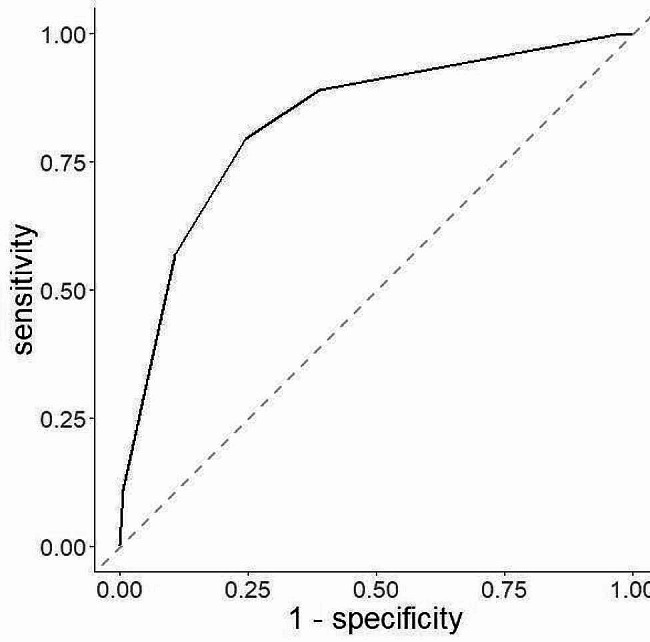
ROC plot with pH as an independent variable and dominant microbiota as a dependent variable. The horizontal axis represents 1 – specificity, while the vertical axis represents sensitivity. The AUC for the dominant microbiota was 0.81

Our results indicated that the sensitivity and specificity of H_2_O_2_-indicating *Lactobacillus* morphotypes were 91.3% and 25.28%, respectively (Table 4), while those of pH-indicating *Lactobacillus* morphotypes were 88.09% and 59.52%, respectively (Table 4). The sensitivity and specificity of H_2_O_2_-indicating dominant microbiota were 91.3% and 25.3%, respectively (Table 5), while those of pH-indicating dominant microbiota were 86.27% and 64.45%, respectively (Table 5). NAG sensitivity and specificity for VVC were 40.64% and 84.8%, respectively (Table 6). GUS sensitivity to AV was low at 0.52%, while its specificity was high at 99.93% (Table 7). LEU sensitivity and specificity for AV were 94.73% and 27.49%, respectively (Table 7). Finally, SNA sensitivity and specificity for BV were 80.72% and 96.78%, respectively (Table 8). We used a ROC curve to determine whether pH is an accurate indicator of *Lactobacillus* morphotypes and the dominant microbiota. As shown in Figs. 2 and 3, the areas under the curve (AUC) of the ROC curves were greater than 0.8, which is an acceptable value.

**Table 4:** Comparison of the two testing methods: morphological testing( Lactobacillus morphotypes)and functional testing (H_2_O_2,_ pH)

	Lactobacillus morphotypes				
		Abnormal	Normal	%Sensitivity	%Specificity
H_2_O_2_	Abnormal	1081	2778	91.3	25.28
	Normal	103	940		
pH	Abnormal	1043	1505	88.09	59.52
	Normal	141	2213		

^a^*Lactobacillus* morphotypes: 30 or more morphotypes per oil immersion was considered normal, and fewer than 30 morphotypes per oil immersion was considered abnormal

^b^ H_2_O_2_: In the vagina, H_2_O_2_ restricts the growth of most pathogens. According to the technical parameters of the testing kit, a H_2_O_2_ concentration of less than 3.0 µmol/L was considered abnormal

^c^ pH: Female vaginal pH is maintained at 3.8 to 4.5; anything above 4.5 was considered abnormal.

**Table 5:** Comparison of the two testing methods: morphological testing (dominant microbiota) and functional testing (H_2_O_2,_pH)

	Dominant microbiota				
		Abnormal	Normal	%Sensitivity	%Specificity
H_2_O_2_	Abnormal	1081	2777	91.3	25.3
	Normal	103	941		
pH	Abnormal	1370	1178	86.27	64.45
	Normal	218	2136		

^a^ Dominant microbiota: When the largest biomass density in the microbiota was *Lactobacillus*, this was considered normal, otherwise it was considered abnormal

^b^ H_2_O_2_: In the vagina, H_2_O_2_ restricts the growth of most pathogens. According to the technical parameters of the testing kit, a H_2_O_2_ concentration of less than 3.0 µmol/L was considered abnormal

^c^ pH: Female vaginal pH is maintained at 3.8 to 4.5; anything above 4.5 was considered abnormal.

**Table 6:** Comparison of the two testing methods: morphological testing (VVC) and functional testing (NAG)

	VVC				
		Abnormal	Normal	%Sensitivity	%Specificity
NAG	Abnormal	217	664	40.64	84.8
	Normal	317	3704		

^a^ NAG: A concentration of NAG above 6.0 U/L was considered abnormal

^b^ VVC: When budding yeasts or pseudohyphae were found under optical microscopy, potentially indicating VVC, this was considered abnormal; otherwise, it was considered normal.

**Table 7:** Comparison of the two testing methods: morphological testing (AV) and functional testing (GUS, LEU)

	AV				
		Abnormal	Normal	%Sensitivity	%Specificity
GUS	Abnormal	10	2	0.52	99.93
	Normal	1906	2984		
LEU	Abnormal	1815	2165	94.73	27.49
	Normal	101	821		

^a^ AV: A Donders score ≥ 3 was considered abnormal

^b^ GUS: A GUS concentration above 16.0 U/L was considered abnormal

^c^ LEU: A LEU concentration above 9.0U/L was considered abnormal.

**Table 8:** Comparison of the two testing methods: morphological testing (Nugent) and functional testing (SNA)

	Nugent				
		Abnormal	Normal	%Sensitivity	%Specificity
SNA	Abnormal	268	147	80.72	96.78
	Normal	64	4423		

^a^ Nugent Score is the “gold standard” for laboratory diagnosis of BV; a total score of 0 to 3 was considered normal; ≥7 was diagnosed as BV

^b^ SNA: An SNA concentration above 7.0 U/L was considered abnormal.

## Discussion

During a woman’s reproductive years, *Lactobacillus*, estrogen, and vaginal pH play a crucial role in maintaining the vaginal microbiome balance [10]. The vaginal microbiome can be clustered into five community state types: *Lactobacillus crispatus* (CST-I),*Lactobacillus iners* (CST-III),*Lactobacillus gasseri* (CST-II), *Lactobacillus jensenii* (CST-V), and CST-IV (*Gardnerella*, *Atopobium*, *Mobiluncus*, *Prevotella* and other taxa in the order Clostridiales) [11, 12]. The normal vaginal microbiome of most women produces lactic acid and H_2_O_2_, which restrict the growth of most pathogens and help maintain a low and protective pH (3.5–4.5) [13]. This may explain why pH showed high sensitivity to the dominant microbiota and *Lactobacillus* morphotypes of 86.27% and 88.09%, respectively. Our findings were consistent with lactic acid, at a sufficiently acidic pH, being a potent microbicide, and lactic acid produced by vaginal lactobacilli may help protect against reproductive tract infections [14]. The pH value can be measured by a clinician at the bedside using precision pH test paper, which is the best method for obtaining an accurate pH value [15]. In this study, we performed pH value measurements with an LTS-V800 (Zhuhai Lituo Biotechnology Co., Ltd). We found that the AUC of the ROC was greater than 0.8 for both *Lactobacillus* morphotype and the dominant microbiota. Therefore, the LTS-V800 system was a convenient and feasible means of pH measurement.

Significantly, we also found that H_2_O_2_ had high sensitivity to the dominant microbiota and *Lactobacillus* morphotypes, exhibiting 91.3% and 91.3% sensitivity, respectively. Conversely, H_2_O_2_ specificity for the dominant microbiota and *Lactobacillus* morphotypes was relatively low, showing 25.3% and 25.28% specificity, respectively. One reason for these unsatisfactory specificity may be that *Lactobacilli* produce little or no H_2_O_2_ under hypoxic conditions or that H_2_O_2_ is inactivated by the powerful antioxidant effect [13].

It is well established that when the vaginal microbiome is characterized by low concentrations of, or an absence of, *Lactobacilli* and elevated concentrations of pathogenic microorganisms, reproductive tract infections occur. Based on this, a vaginal microbiota evaluation method has been developed to rapidly diagnose common vaginal inflammatory diseases [16]. Although vaginal microbiota evaluations can be helpful, they are time-consuming and require expert operators. Furthermore, as a result of the uneven distribution of medical resources in China, vaginal microbiota evaluations may not be available in community hospitals [7].

More convenient methods are therefore needed to diagnose reproductive tract infections in community hospitals, and functional testing is worth considering. The Nugent score is widely considered the reference standard laboratory method for diagnosing BV [17]. The pathogenic microorganisms causing BV, such as *G. vaginalis*, utilize sialidase to support the degradation, foraging, and depletion of protective host mucus barriers [18] and modify the immune response [19]. Our results showed that SNA sensitivity and specificity for BV were 80.72% and 96.78%, respectively, and multiple point-of-care tests, such as the Osom BV Blue test (Sekisui Diagnostics), are available for BV diagnosis [17].

Currently in our laboratory, a diagnosis of VVC requires a combination of clinical findings and Gram staining of vaginal discharge to indicate budding yeasts, hyphae, or pseudohyphae [15]. It is reported that β-N-acetylglucosaminidase (NAG) can degrade host glycoproteins to aid the invasion of pathogenic fungi [20]. In this study, NAG specificity for VVC was 84.8%. These results were consistent with Candida species secreting several hydrolytic enzymes, which play an important role in adhesion, tissue penetration,invasion and the destruction of host tissues [21]. Inflammation may not be a clinical symptom of VVC, but it is the cardinal feature of AV [10]. LEU is widely used to indicate the presence of inflammation, and we showed that LEU sensitivity and specificity for AV were 94.73% and 27.49%, respectively. Based on the commercial diagnostic kit, GUS correlates with AV. Wang et al. reported that when samples tested positive for H_2_O_2_, LE, GUS, or coagulase, or both GUS and coagulase, AV could be diagnosed. Whereas, among AV patients, there was a low detection rate of Group B *Streptococcus*, and such patients rarely tested positive for GUS [22]. Our result showed a similar low rate of Group B *Streptococcus* detection of 0.626% (12/1916). Although during inflammation, GUS participates in tissue injury [23], we showed the high specificity (99.93%) and low sensitivity (0.52%) of GUS for AV. Therefore, further fundamental research into functional testing is required.

Some functional tests, such as PH, H_2_O_2_, SNA and LEU, showed high sensitivity to morphological tests. A limitation of morphological tests is their poor sensitivity. Amsel has a sensitivity of 60–72% for the diagnosis of BV [17]. Gram staining has a sensitivity of up to 65% for the diagnosis of VVC [17]. These functional tests can help alert to the need to review morphological tests, thereby complementing morphological tests. However, Wu et al. reported that the inspection of five vaginitis indexes is simpler and provides greater accuracy, as well as a more comprehensive overview of the microorganisms present in the vagina [7]. In their opinion,functional tests are analyzed in their entirety, without considering the individual clinical significance of each enzyme component [7]. Unlike molecular tests, which can facilitate the accurate detection of vaginitis [24], most functional tests do not diagnose vaginitis specifically; for example, pH, H_2_O_2_, and LEU showed poor specificity. There is no specific one-to-one correspondence between hydrolytic enzymes and pathogens. Leucocyte esterase, for example, can be present in any inflammatory response, not just in aerobic vaginitis [25]. The specificity of NAG and GUS was high, but their sensitivity was low, at 40.64% and 0.52%, respectively. When we combined two functional tests with high sensitivity or specificity, such as LEU and GUS, this aided the identification of AV. Optimizing different functional tests can therefore help diagnose vaginitis in community hospitals. While molecular tests are costly and can give false positives [24], functional tests have limitations that should also be noted. Sample collection, sample elution, sample loading, data readout, and blood samples all affect functional testing. Therefore, we should implement strict quality controls based on manufacturer specifications. To obtain satisfactory functional test results, in some cases human visual interpretation is also required. Further investigations should be carried out of functional tests with greater sensitivity and specificity, along with the optimization of different functional test schemes, to accurately rule out infections in the reproductive tract.

## Conclusion

In this study, we revealed that the sensitivity of certain functional tests (pH, H_2_O_2_, SNA, LEU) in identifying vaginal inflammatory diseases was satisfactory to inform clinicians regarding the need for morphological tests, but their specificity was currently insufficient. By contrast, NAG and GUS showed excellent specificity in identifying vaginal inflammatory diseases, but their sensitivity was limited. Therefore, we were unable to provide a satisfactory functional test scheme at present. Future studies will investigate functional tests with greater sensitivity and specificity to accurately rule out reproductive tract infections. Morphological tests are currently considered the primary reference method when functional and morphological test results are inconsistent.

## Data Availability

Data sets used and/or analyzed during this study are available from the corresponding authors upon reasonable request.
